# Reviewer acknowledgement 2015

**DOI:** 10.1186/s13098-016-0132-x

**Published:** 2016-03-02

**Authors:** Daniel Giannella-Neto

**Affiliations:** Universidade Nove de Julho, São Paulo, Brazil

## Contributing reviewers

The Editors of *Diabetology and Metabolic Syndrome* would like to thank all of our reviewers, both external and Editorial Board Members, who have contributed to the journal in Volume 7 (2015) and whose valuable support is fundamental to the success of the journal.

**Mohsen Agharazii**

Canada

**Catherine Arden**

UK

**Jean-Luc Ardilouze**

Canada

**Benedikt Aulinger**

Germany

**Athina Bakopoulou**

Greece

**Andrea Baragetti**

Italy

**Andrew Bateman**

Canada

**Cecília Berardi**

USA

**Eliete Bouskela**

Brazil

**Laima Brazionis**

Australia

**Amelia Brunani**

Italy

**Cleber Camacho**

Brazil

**Ana Valeria Castro**

Brazil

**Krzysztof Celinski**

Poland

**Benjamin G. Challis**

UK

**Weihong Chen**

China

**Andriy Cherkas**

Ukraine

**Vinicius Cruzat**

Australia

**Bianca De Almeida-Pititto**

Brazil

**Katia De Angelis**

Brazil

**Anize Delfino von Frankenberg**

Brazil

**Basset Eiessawy**

USA

**Aline Fofonka**

Brazil

**André Fragoso**

Portugal

**Clemens Fürnsinn**

Austria

**Anel Gómez García**

USA

**Sandra Roberta Gouvea Ferreira**

Brazil

**Jorge Gross**

Brazil

**Junling Han**

China

**Robert Hegele**

Canada

**Elizabeth Holmes-Truscott**

Australia

**Paul Holvoet**

Belgium

**Qin Huang**

China

**Daniela Inoue**

Brazil

**Shirin Jahan Mumu**

Bangladesh

**Peng Juan**

China

**Sanjay Kalra**

India

**Ahsan Khandoker**

Australia

**Attila Kiss**

Austria

**Aloysius Klingelhutz**

USA

**Andrzej Krolewski**

USA

**Andrea Leiva**

Chile

**Duo Li**

China

**Xin Li**

China

**Zhuofeng Lin**

China

**Heno Lopes**

Brazil

**Usman Malabu**

Australia

**Marcio C. Mancini**

Brazil

**Isabelle Marc**

Canada

**Elaine Marqueze**

Brazil

**Alejandra de Moreno de LeBlanc**

Argentina

**Richelle Mychasiuk**

Canada

**Carolina Navarro-Blanco**

Spain

**Arnold Ng**

Australia

**Georges Nguefack-Tsague**

Cameroon

**Alexia Pena**

Australia

**Angela Pirillo**

Italy

**Roberto Pontarolo**

Brazil

**Alex Rafacho**

Brazil

**Andre Reis**

Brazil

**Maria Elizabeth Rossi da Silva**

Brazil

**Beatriz Schaan**

Brazil

**Yujuan Shan**

China

**David Sigalet**

Canada

**Akhilesh Tamrakar**

India

**Ivani Trombetta**

Brazil

**Bart Van der Schueren**

Belgium

**Sergio Vencio**

Brazil

**Glauce Viana**

Brazil

**Greisa Vila**

Austria

**Adam Watkins**

UK

**Xinhua Xiao**

China

**Xiaoyan Zhou**

China

**Yu-jie Zhou**

China

